# ANK, a Host Cytoplasmic Receptor for the *Tobacco mosaic virus* Cell-to-Cell Movement Protein, Facilitates Intercellular Transport through Plasmodesmata

**DOI:** 10.1371/journal.ppat.1001201

**Published:** 2010-11-18

**Authors:** Shoko Ueki, Roman Spektor, Danielle M. Natale, Vitaly Citovsky

**Affiliations:** Department of Biochemistry and Cell Biology, State University of New York, Stony Brook, New York, United States of America; Ohio State University, United States of America

## Abstract

Plasmodesma (PD) is a channel structure that spans the cell wall and provides symplastic connection between adjacent cells. Various macromolecules are known to be transported through PD in a highly regulated manner, and plant viruses utilize their movement proteins (MPs) to gate the PD to spread cell-to-cell. The mechanism by which MP modifies PD to enable intercelluar traffic remains obscure, due to the lack of knowledge about the host factors that mediate the process. Here, we describe the functional interaction between *Tobacco mosaic virus* (TMV) MP and a plant factor, an ankyrin repeat containing protein (ANK), during the viral cell-to-cell movement. We utilized a reverse genetics approach to gain insight into the possible involvement of ANK in viral movement. To this end, ANK overexpressor and suppressor lines were generated, and the movement of MP was tested. MP movement was facilitated in the ANK-overexpressing plants, and reduced in the ANK-suppressing plants, demonstrating that ANK is a host factor that facilitates MP cell-to-cell movement. Also, the TMV local infection was largely delayed in the ANK-suppressing lines, while enhanced in the ANK-overexpressing lines, showing that ANK is crucially involved in the infection process. Importantly, MP interacted with ANK at PD. Finally, simultaneous expression of MP and ANK markedly decreased the PD levels of callose, β-1,3-glucan, which is known to act as a molecular sphincter for PD. Thus, the MP-ANK interaction results in the downregulation of callose and increased cell-to-cell movement of the viral protein. These findings suggest that ANK represents a host cellular receptor exploited by MP to aid viral movement by gating PD through relaxation of their callose sphincters.

## Author Summary

During infection, plant viruses utilize their cell-to-cell movement proteins (MPs) to gate plant intercellular connections, the plasmodesmata (PD), and spread between the host cells. The mechanism by which MPs facilitate their cell-to-cell translocation remains elusive. We have identified a tobacco ankyrin repeat-containing protein, ANK, that interacts with MP of *Tobacco mosaic virus* (TMV) both *in vivo* and *in vitro*. When coexpressd with MP, ANK promoted intercellular transport of MP and, by implication, gated PD for its movement and facilitated the viral spread. Conversely, RNAi-based knock-down of the ANK gene expression resulted in reduced MP movement and attenuated viral infection. Coexpression of ANK with MP resulted in reduction of callose deposits at PD, which are known to function as PD channel sphincters. Interestingly, expressing ANK alone did not affect callose deposits. Thus, ANK most likely represents a cellular factor that recognizes MP and acts synergistically with it to gate PD and mediate MP transport through these channels.

## Introduction

Plasmodesma (PD) is a cell-wall spanning channel that interconnects plant cells and is unique for plants. This structure is characterized by a neck-like constriction at its each end, is lined by the plasma membrane, and is traversed by a strand of appressed endoplasmic reticulum, providing endomembrane and cytoplasmic connections to adjacent cells. PD mediate direct macromolecular exchange between the connected cells, and this transport is highly regulated (for review, see [1], [2], [3]).

PD are also utilized by plant viruses for infection. After initial inoculation and replication in the infected cell, plant viruses spread to the neighboring cells and throughout the entire plant. For this transport, viruses utilize their cell-to-cell movement proteins, MPs [3], [4], [5], [6], [7], [8], [9]. The traffic of viral MPs represents a paradigm and a model system for macromolecular transport through PD. For example, *Tobacco mosaic virus* (TMV) MP, the archetype of many viral MPs, presumably associates with the viral genomic RNA to form a movement ribonucleocomplex [10], targets this complex to PD [11], [12], [13], [14], and increases the PD size exclusion limit to translocate the movement complex through the PD channel [15], [16].

To date, two cellular factors, actin filaments and callose have been implicated in control of transport through PD [17], [18], [19], [20], [21], [22], [23], [24]. A recent study suggested that the *Cucumber mosaic virus* and TMV MP may sever the actin filaments to aid their cell-to-cell transport [25], and callose, which accumulates at the neck region of PD [18], represents a molecular sphincter that restricts cell-to-cell transport of macromolecules [18], [19], [20], [21], [22], [23], [24]. The level of callose in the cell wall is primarily determined by a balance between the enzymatic activities of callose synthases and β-1,3-glucanases [26], [27], [28]. Callose deposits presumably affect transport through PD, because their elevated accumulation delays local and systemic movement of different viruses [21], [22], [23], [24]. Thus, it would make a biological sense if plant viruses had evolved a mechanism to regulate, via viral MPs and as yet unknown host factor(s), callose deposits to allow their own movement through PD. So far, however, the existence of such a mechanism has not been demonstrated.

To date, several and very diverse host proteins have been shown to bind viral MPs. For example, cytoskeletal elements, calreticulin, pectin methylesterases, and DnaJ chaperones have all been shown to interact with TMV MP [11], [29], [30], [31], [32], [33], [34]. Yet, none of these factors had any effects on callose deposits at PD. The role of one class of host proteins, ankyrin repeat-containing proteins (ANKs) — also known as TIP1-3, and initially reported to bind MP of the *Potato virus X* (PVX) *in vitro* [35] — in viral movement has received no attention since their discovery. Here, we investigated the potential involvement of ANK in regulation of mobility of TMV MP through PD. Our data indicate that ANK interacts with MP and promotes reduction in PD callose deposits and subsequent MP cell-to-cell transport.

## Results

### ANK promotes MP cell-to-cell movement

**Figure 1:**
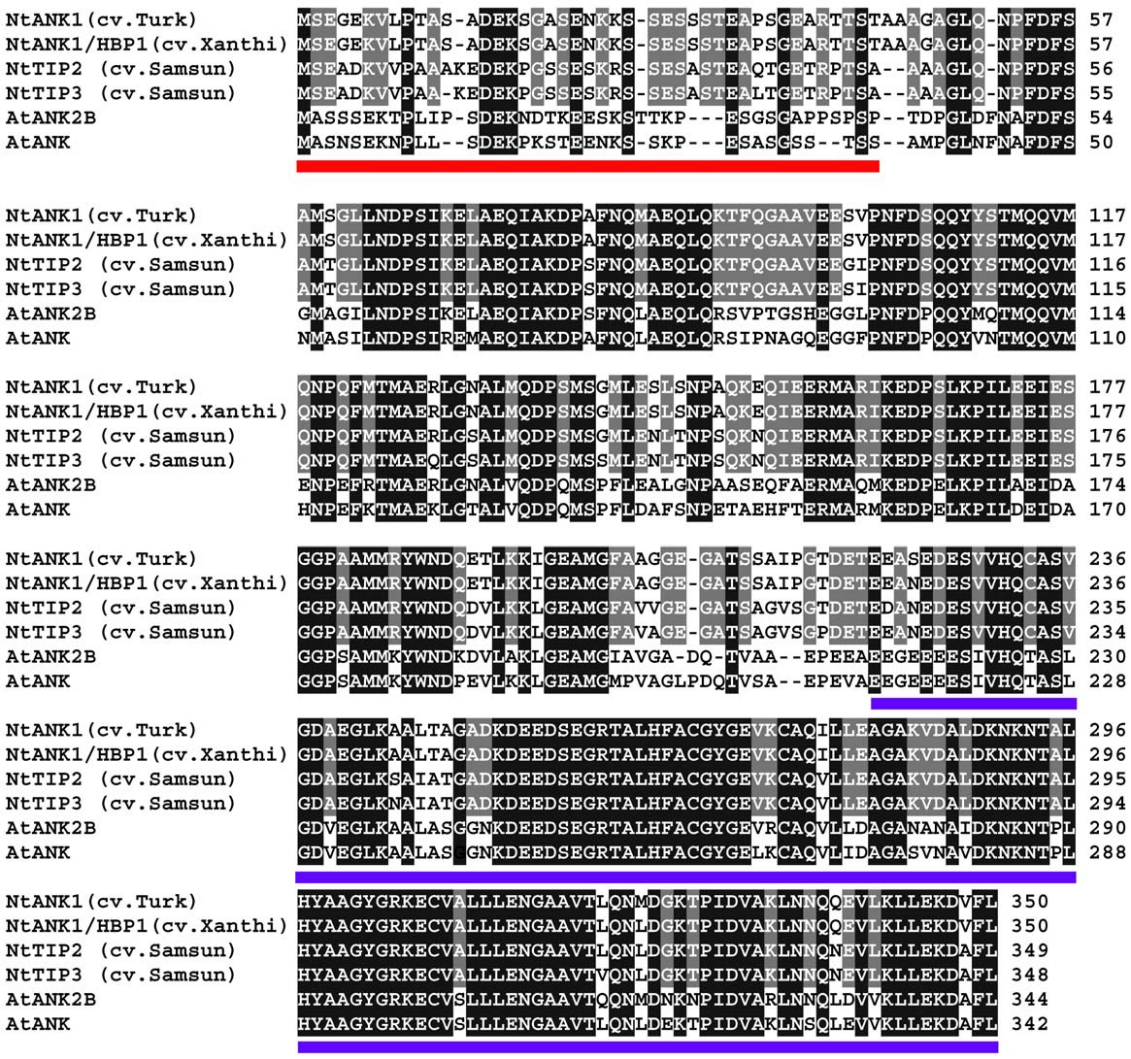
Amino acid sequence alignment of ANK proteins from different tobacco cultivars and Arabidopsis. The ANK from *N. tabacum* cv. Turk (accession number GU320195) was aligned using the T-Coffee program (http://www.ebi.ac.uk/Tools/t-coffee/index.html) with its closest homologs from *N. tabacum* cv. Xanthi (*Nt*ANK1/HBP1, accession number AAK18619/AAN63819), *N. tabacum* cv. Samsun (*Nt*TIP2 and *Nt*TIP3, accession numbers AAO91861.1 and AAO91862.1, respectively), and *Arabidopsis thaliana* (*At*AKR2B and *At*AKR2, accession numbers NP_179331 and NP_849498, respectively). Note that *Nt*TIP1 from *N. tabacum* cv. Samsun is identical to NtANK1/HBP1 [35]. Amino acid residues identical between tobacco ANKs are highlighted in gray and between all ANK homologs are highlighted in black. The PEST and ankyrin repeat-containing domains are underlined in red and blue, respectively.

To evaluate TMV spread within infected plant tissues, it is useful to employ a plant host that does not develop necrosis upon infection with this virus. Thus, we chose the cultivar of tobacco (*Nicotiana tabacum cv*. Turk), which lacks the *N*-gene and, therefore, does not produce a hypersensitive cell death response to TMV [22], [36], [37]. Because the ANK homolog of this cultivar has not been isolated, we cloned its cDNA and compared the predicted amino acid sequence with several known ANK homologs from tobacco and Arabidopsis. Figure 1 shows that the ANK sequence is well conserved in these plants, with the highest degree of identity found in the C-terminal ankyrin repeats (underlined in blue), which represent the protein domains typically involved in protein-protein interactions [38], [39]. N-termini of ANK proteins also carry a loosely-defined PEST domain (underlined in red) [35], [40], [41], which often, but not always, serves as signal for proteolysis [42], [43], [44].

We then utilized reverse genetics to gain the first insight into the possible involvement of ANK in viral movement. We generated transgenic tobacco plants with RNAi-based suppression of the *ANK* gene. For specific suppression, we targeted the sequences of *ANK* that encode the N-terminal part of the protein, rather than its more conserved C-terminal ankyrin motifs (Figure S1). Based on quantitative real time PCR (qPCR) analysis of several independently-transformed lines, we identified severe and moderate suppressors, in which the *ANK* gene expression levels were reduced to 5% and 15–40%, respectively, of the wild-type expression level (Figure S2A). The severe suppressors also produced a markedly chlorotic phenotype (line RNAi ANK3, Figure S2B), consistent with the known involvement of ANK homologs in chloroplast biogenesis [45]. The moderate suppressors, however, appeared healthy and did not develop any detectible morphological or developmental phenotypes (line RNAi ANK1, Figure S2B). Two such transgenic lines, RNAi ANK1 and RNAi ANK2, were selected for further analyses.

First, to confirm the specificity of the RNAi-based ANK suppression, we examined the expression levels of two genes, *ASPARTATE AMINOTRANSFERASE* (*AATF*) and *MAGNESIUM PROTOPORPHYRIN IX (MgPP)*, which are unrelated to ANK, but contain a short, 17-bp nucleotide sequence that is also found in the *ANK* sequence used for the RNAi suppression (Figure S1). No differences were detected in the expression levels of *AATF* and *MgPP* in both RNAi ANK lines and in the wild type plants (Table S1), indicating that the *ANK* suppression in our RNAi ANK lines was specific.

**Figure 2:**
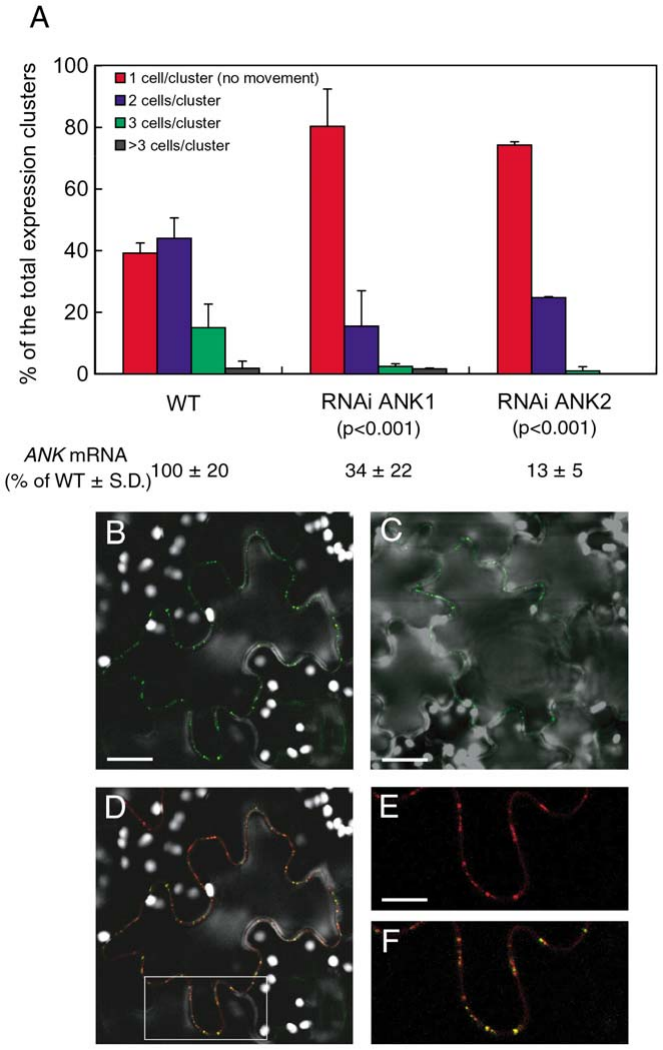
Reduced cell-to-cell movement of MP-YFP in RNAi ANK1 and RNAi ANK2 plants and colocalization of MP-YFP and PDCB-mCherry. (A) Quantification of MP-YFP movement and *ANK* transcript levels. Standard deviations (S. D.) and the unpaired two tailed Student's *t-*test P-values for statistical significance of difference between the RNAi ANK1, RNAi ANK2, and wild-type (WT) plants are indicated. (B–F) Colocalization of MP-YFP and PDCB-mCherry to PD in RNAi ANK1 plants. Panels B, C represent YFP signal and show PD localization of MP-YFP in RNAi ANK1 and wild-type plants, respectively. Bars  = 50 µm. Panel D represents merged YFP and mCherry signals and shows colocalization of MP-YFP and PDCB-mCherry in the same cell as in panel B. The area outlined by a white rectangle in panel D is presented at a larger magnification in panels E, F, showing PD localization of PDCB-mCherry and PD colocalization of PDCB-mCherry and MP-YFP, respectively. Bars  = 25 µm. Note that MP/PDCB colocalization is not complete because MP preferentially localizes only to secondary PD [13]. YFP signal is in green, mCherry signal is in red, overlapping YFP/mCherry signal is in yellow, and plastid autofluorescence is in white. All images are single confocal sections.

We then used RNAi ANK1 and RNAi ANK2 to examine the effect of the reduction in the endogenous *ANK* expression on MP cell-to-cell movement. To this end, MP was tagged with YFP and the encoding constructs introduced into the wild-type and RNAi transgenic lines by microbombardment. The MP-YFP movement was determined by confocal microscopy two days after bombardment. Figure 2A shows that, in the wild-type plants, approximately 60% of the signal was distributed between 2 to 3 cells. These observations were consistent with the known rate of the MP movement in plant tissues [46], [47], and typical for this tobacco cultivar. In contrast, 70–75% of the signal remained confined to a single cell in RNAi ANK1 and RNAi ANK2 lines, indicating substantial decrease in the MP movement. Statistical evaluation of these data by the unpaired two-tailed Student's *t*-test confirmed that the MP movement capacities in the RNAi ANK1 and RNAi ANK2 plants were similar to each other, but different from those in the wild-type plants (Figure 2A). MP was found associated with PD in a punctate pattern characteristic for PD localization in both RNAi ANK1 (Figure 2B, D–F) and in RNAi ANK2 plants (not shown), as in wild-type (Figure 2C) [46], [48], [49], [50], [51]. Importantly, the MP colocalized with a PD marker, the PD callose binding protein (PDCB) [52] (Figure 2D–F), further confirming the targeting of the viral protein to PD in RNAi ANK background. MP localization to PD was also confirmed in both wild-type and RNAi ANK backgrounds at different time periods after bombardment (Figure S3A, B, D, E); furthermore, the expression levels of MP-YFP in these plants were essentially the same, indicating that the alteration of the ANK amount in the cell does not affect the accumulation of MP (Figure S3G). Thus, the ANK suppression most likely affected MP translocation through PD, rather than its PD targeting or level of expression.

MP is known to associate with the host cell ER [53], [54], [55], [56], [57], [58]. Thus, we examined whether ANK is also involved in cell-to-cell transport of ER-associated plant proteins. Specifically, we tested the cell-to-cell diffusion of two of such proteins, the Arabidopsis calnexin (CNX) and calmodulin-regulated Ca^2+^-ATPase ER membrane protein (ACA2), which move between cells presumably due to membrane diffusion through the PD-spanning ER [53]. Table 1 shows no statistically significant effects of ANK suppression on this cell-to-cell diffusion of YFP-tagged CNX and ACA2 as well as free YFP that diffuses through cytoplasm. Thus, the effect of ANK on the MP movement is specific.

**Table 1:** Cell-to-cell diffusion of CNX and ACA2 in wild-type and RNAi ANK1 and RNAi ANK2 plants.

	Plant lines^A^	% of the total signal clusters			
		1 cell/cluster	2 cells/cluster	3 cells/cluster	P-value^C^
**CNX-YFP**	WT (114^B^)	64.9	33.3	1.8	
	RNAi ANK1 (120)	67.5	30.0	2.5	> 0.1
	RNAi ANK2(120)	60	38.3	1.7	> 0.1
**ACA2-YFP**	WT (129)	66.7	31.0	2.3	
	RNAi ANK1 (141)	66.7	30.5	2.8	> 0.1
	RNAi ANK2 (133)	60.2	38.3	1.5	> 0.1
**Free YFP**	WT (153)	82.3	17.7	0	
	RNAi ANK 1 (142)	80.3	19.7	0	> 0.1
	RNAi ANK 2 (144)	84.7	15.3	0	> 0.1

A WT, wild-type.

B The numbers of analyzed clusters are shown in parentheses.

C P-values were calculated using the unpaired two tailed Student *t-*test. They show no statistically significant differences between movement of the tested proteins in the RNAi ANK transgenic lines and in the wild-type plants.

**Figure 3:**
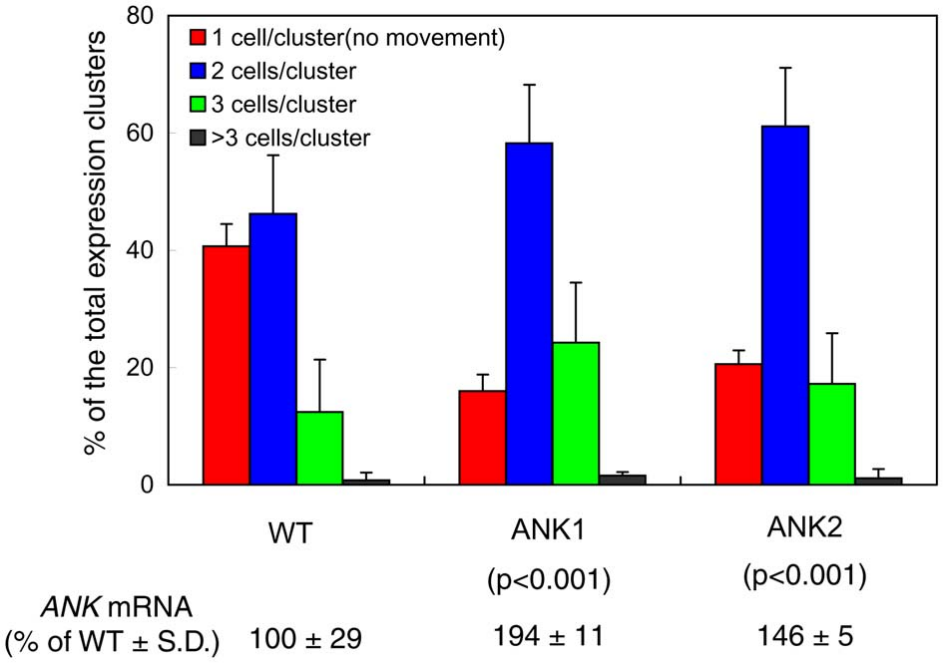
Enhanced cell-to-cell movement of MP-YFP in ANK1 and ANK2 plants. Quantification of MP-YFP movement and *ANK* transcript levels is shown with standard deviations (S. D.) and the unpaired two tailed Student's *t-*test P-values for statistical significance of difference between the ANK1 or ANK2 and wild-type (WT) plants.

That the lack of ANK negatively affects MP movement is suggestive of ANK's involvement in this transport process. To support this notion further, however, it is useful also to demonstrate that overproduction of ANK enhances movement. To this end, we generated another series of transgenic lines, this time, expressing *ANK* under the control of a constitutive promoter. We then selected two of these lines, ANK1 and ANK2, which exhibited moderate levels of the *ANK* transcript overexpression (1.5–2 fold, see Figure 3). We could not measure directly the levels of the ANK protein in these lines due to the lack of a specific anti-ANK antibody. Instead, we produced transgenic lines overexpressing ANK tagged with a short (1 kDa) StrepII epitope [59], [60] and demonstrated close correlation between the levels of the *ANK-StrepII mRNA* and the ANK protein (Figure S4), suggesting that the increased levels of *ANK* transcription lead to the increased accumulation of ANK protein.

When the ANK1 and ANK2 plants were examined for their ability to support cell-to-cell movement of MP-YFP, the movement was enhanced in both lines. Specifically, whereas 58% of the signal moved in the wild-type plants, the extent of movement in the ANK transgenic plants reached a total of 84–86% (Figure 3). In addition, the extent of the movement was also slightly, but consistently enhanced, with 7% of the signal found in three cell-clusters in the wild-type tissues, and in 11–17% in the ANK1 and ANK2 plants. The unpaired two-tailed Student's *t-*test confirmed the statistical significance of the differences in cell-to-cell movement of MP between the wild-type and the ANK transgenic plants (Figure 3). As shown in Figure S3, the PD localization pattern or the accumulation level of MP were not affected in these plants. Collectively, these data indicate that the ANK may represent a plant factor that facilitates the MP cell-to-cell movement.

### ANK is required for TMV infection

**Figure 4:**
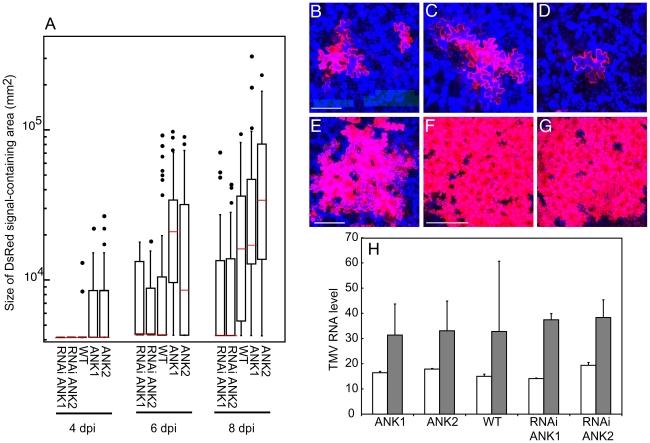
Local movement of TMV-DsRed is facilitated in ANK1 and ANK2 plants and delayed in RNAi ANK1 and RNAi ANK2 plants. (A) Quantification of TMV-DsRed local movement in the indicated plant lines based on the size of DsRed signal-containing areas at 4, 6, and 8 days post inoculation (dpi). The box-and-whisker plots show the statistical distribution of the data, with horizontal red lines across the boxes representing the median size of the DsRed expression foci, the ends of boxes indicating the quartile results, and the circles representing outliers. Statistical significance of differences between each transgenic line and the wild-type (WT) plants was analyzed by ANOVA and calculated as P<0.0001. (B–G) TMV-DsRed signal in WT plants at 4 and 6 (panels B and C, respectively), RNAi ANK1 plant at 6 dpi (panel D), ANK2 plant at 6 dpi (panel E), WT plant at 10 dpi (panel F) and RNAi ANK1 plant at 15 dpi (panel G). Bars  = 100, 200, 400 µm for panels B–D, E, F, respectively. DsRed signal is in red. All images are single confocal sections. (H) Quantification of TMV-DsRed replication efficiency based on the levels of TMV RNA in protoplasts prepared from the indicated plant lines at 16 and 40 h post inoculation (hpi) (open and shaded bars, respectively). The shown values were normalized to the amounts of *TUBLIN* transcript in the same samples.

Biologically, suppression of MP mobility should lead to delayed infection, while increase of the MP trafficking through PD is expected to enhance the viral spread. To examine this possibility, we inoculated the ANK overexpressor and suppressor lines as well as the wild type plants with a recombinant virus that carries an autofluorescent tag DsRed (TMV-DsRed) in the place of its coat protein. The local movement of this virus was detected by the appearance and spread of the DsRed signal. Figure 4 shows that whereas the viral transport became visible in all plants approximately at the same time after inoculation (panels A, B), the subsequent viral spread occurred faster in the ANK1 and ANK2 lines than in the wild type plants (panels A, C, E). In contrast, in RNAi ANK1 and RNAi ANK2 lines, the virus moved significantly slower than in wild type plants (Figure 4A, C, D). Importantly, the viral movement in the ANK suppressor lines was delayed, but not arrested, and it reached the wild-type levels late in the infection process (Figure 4F, G). It is important to assertain whether the positive effect of ANK overexpression and negative effect of ANK suppression on the viral movement are not due to increased or decreased replication of the virus in the ANK overexpressor or suppressor lines, respectively. To this end, we produced protoplasts from ANK1, ANK2, RNAi ANK, RNAi ANK2, and wild type plants and infected them with TMV-DsRed. Figure 4H shows that no significant differences were detected in the replication levels of the virus between any of the tested plant lines, suporting the idea that the effect of ANK on the spread of the virus was due to its *bona fide* effect on the cell-to-cell movement capacity of the viral MP.

### MP and ANK directly interact at PD

**Figure 5:**
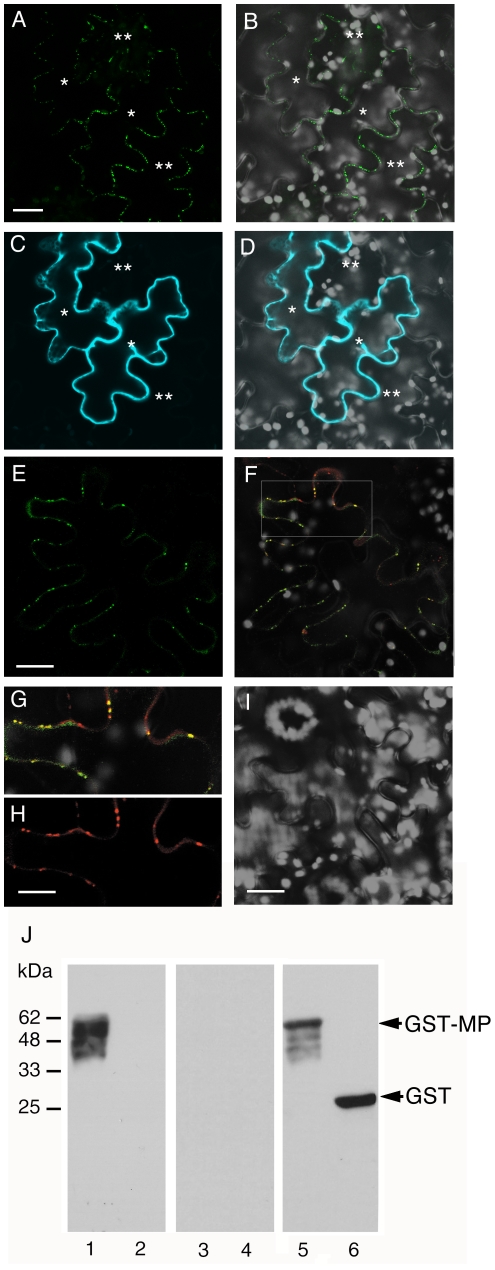
Subcellular localization of MP and ANK and interaction between MP and ANK *in planta* and *in vitro*. (A, B) PD localization of MP-YFP coexpressed with CFP-ANK. (C, D) Cytoplasmic localization of CFP-ANK coexpressed with MP-YFP. Single asterisks indicate initially expressing cells, and double asterisks indicate cells to which MP-YFP has moved. Panels A, C represent YFP and CFP signals, respectively, and panels B, D represent merged images of YFP (in green) or CFP (in blue) and plastid autofluorescence (in white) signals. Images are projections of three confocal sections. Bars  = 20 µm. (E–H) BiFC assay for the MP-ANK interaction and colocalization of MP-cYFP/nYFP-ANK complexes and PDCB-mCherry *in planta*. Panel E represents YFP signal and shows interaction between MP-cYFP and nYFP-ANK and PD localization of the interacting proteins. Bar  = 20 µm. Panel F represents merged YFP and mCherry signals and shows colocalization of MP-cYFP/nYFP-ANK and PDCB-mCherry in the same cell as in panel E. The area outlined by a white rectangle in panel F is presented at a larger magnification in panels G, H, showing PD colocalization of MP-cYFP/nYFP-ANK and PD localization of PDCB-mCherry, respectively. Bar  = 10 µm. (I) MP-cYFP does not interact with nYFP-NADK3 in the BiFC assay. Bar  = 20 µm. YFP BiFC signal is in green, mCherry signal is in red, overlapping YFP/mCherry signal is in yellow, and plastid autofluorescence is in white. Images are single confocal sections. (J) Renatured gel blot overlay assay for the MP-ANK interaction *in vitro*. Lane 1, GST-MP + ANK-StrepII; lane 2, GST + ANK-StrepII; lane 3, GST-MP + NADK3-StrepII; lane 4, GST + NADK3-StrepII. Lanes 1–4 were probed with the anti-StrepII antibody. Lane 5, GST-MP + ANK-StrepII; lane 6, GST + ANK-StrepII. Lanes 5, 6 were probed with the anti-GST antibody. Arrows indicate the positions of GST-MP and unfused GST. The numbers on the left indicate molecular mass standards in thousands of Daltons.

To gain insight into molecular mechanism of the ANK function in MP movement, we first examined the subcellular localizations of both proteins. When MP tagged with YFP and ANK tagged with CFP were transiently coexpressed in tobacco leaves following agroinfiltration, MP displayed the typical punctate pattern of PD localization (Figure 5A, B), whereas ANK was largely cytoplasmic with characteristic transvacuolar strands (Figure 5C, D), consistent with the known cytoplasmic localization of its homologs [41], [45]. Thus, the majority of the ANK population does not colocalize with MP. Furthermore, the presence of ANK did not detectibly alter the MP localization. Indeed, the MP localization pattern in the coexpressing cells (indicated with single asterisks) was essentially identical to that in the neighboring cells (indicated with double asterisks, Figure 5A–D). Taken together with the data in Figure 2 (panels B–F) and Figure S3 (panels A–F), these observations indicate that ANK does not affect PD targeting of MP.

Although the expression pattern of ANK is different from that of MP, MP is known to possesses cytoplasmic domain [56], [58], providing physical basis for potential interaction between a proportion of the ANK population and MP. Thus, we tested whether ANK can directly recognize and bind MP *in vivo*. To this end, we utilized the bimolecular fluorescence complementation (BiFC) assay *in planta* [61]. To date, BiFC represents one of the best assays for protein-protein interactions and subcellular localization of the interacting proteins within living cells. In this approach, proteins are tagged with N-terminal (nYFP) and C-terminal (cYFP) halves of YFP, neither of which fluoresces on its own. Interaction of the tagged proteins results in reconstruction of the YFP signal [62]. In addition, to avoid potentially non-specific effects on protein overexpression on the interaction [63], MP-cYFP was expressed from a relatively weak nopaline synthase promoter [64]. Under these conditions, coexpression of MP-cYFP and nYFP-ANK in tobacco leaf epidermal cells resulted in appearance of strong YFP signal, indicating protein interaction (Figure 5E–G). Importantly, this YFP signal faithfully colocalized with the PDCB-mCherry (Figure 5F–H), clearly demonstrating that the MP-ANK interaction occurs at PD.

The specificity of the MP-ANK interaction was verified in negative control experiments, for which we chose two unrelated cytoplasmic proteins similar in size to ANK (37 kDa), the *Arabidopsis* cytoplasmic NADH kinase (NADK3, 36 kDa) [65], and a fragment of the bacterial ß-glucuronidase (GUS, 37 kDa). Neither NADK3 (Figure 5I) nor the GUS fragment (not shown) promoted reconstruction of the YFP signal when they were tagged with nYFP and coexpressed with MP-cYFP. Thus, the *in vivo* interaction between MP and ANK at PD was specific.

This BiFC data were confirmed by an independent approach, using a renatured gel blot overlay assay for protein interaction [33]. Figure 5J shows that ANK tagged with StrepII specifically interacted with the membrane-immobilized recombinant MP tagged with glutathione S-transferase (GST) (lane 1), whereas no such binding was observed to the immobilized unfused GST (lane 2). Binding of MP to ANK was specific because it did not occurred between MP and NADK3 (Figure 5J, lanes 3, 4) or between MP and CNX or ACA2 (not shown). Thus, MP can specifically recognize and bind ANK both *in vivo* and *in vitro*.

### ANK, in concert with MP, down-regulate callose deposits at PD

**Figure 6:**
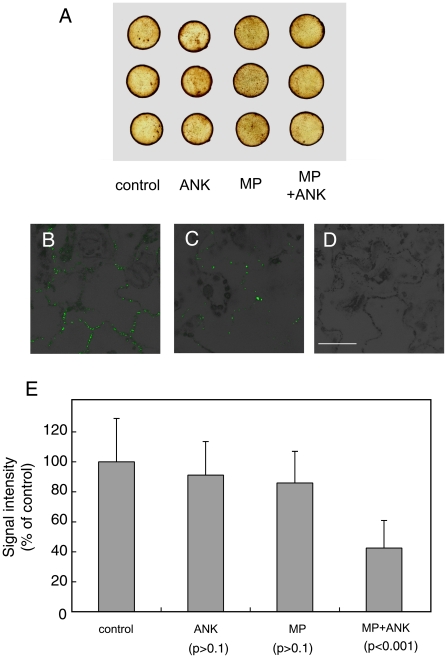
Effects of coexpression of ANK and MP on the content of ROS and callose in plant tissues. (A) Histochemical detection of ROS. (B) Immunodetection of callose deposits at PD in control, mock-transformed plant tissues. (C) Immunodetection of callose deposits at PD following coexpression of ANK and MP. (D) Immunostaining in the absence of the primary, anti-callose antobody in control tissue. Panels B–D represent merged images of the specific signal from callose staining (green) and tissue autofluorescence signal (black and grey). Bar  = 50 µm. (E) Quantification of callose deposits at PD with indicated standard deviations and the unpaired two tailed Student's *t-*test P-values for statistical significance of difference between plants coexpressing ANK and MP and plants expressing separately either ANK or MP or the control plants.

Arabidopsis ANK2 is involved in ROS scavenging through its interaction with ascorbate peroxidase [40], and ROS may, in turn, affect PD transport [66], [67]. Thus, we tested whether expression of ANK alone or together with the MP alters the ROS content of plant tissues. Figure 6A shows histochemical staining for ROS revealed no detectible differences in ROS content between the control, mock-transformed tissues and those expressing ANK or ANK and the MP. Also, the ROS levels in transgenic plants, both ANK overexpressor and suppressor lines, were not significantly different from wild-type lines (Figure S5). Most likely, therefore, ANK facilitates MP movement through the mechanism independent from ROS.

Another possibility is that ANK is involved in regulation of callose sphincters of PD. Callose deposits are known to affect viral movement through PD [20], [21], [22], [23], [24], presumably functioning as a sphincter which physically restricts PD mediated macromolecular trafficking. Thus, ANK may facilitate MP movement by reducing the PD callose deposits. To test this idea, we transiently expressed ANK and MP separately or together with each other in tobacco leaves. The amounts of callose in the expressing tissues were assayed by immunostaining using anti-callose antibody with previously demonstrated specificity [68], [69], followed by quantitative confocal microscopy.

Figure 6 shows that this technique readily visualizes callose deposits in a typical distribution pattern specific for PD with virtually no background signal (panels B and C), and no signal at all in the absence of the primary, anti-callose antibody (panel D). Quantification of the callose-specific signal (Figure 6E) revealed that expression of ANK alone or MP alone resulted in virtually no reduction in callose levels as compared to control tissues. However, when both ANK and MP were coexpressed in the same tissues, the callose-specific signal decreased substantially (Figure 6C, E), to as low as 37% of the control. These results were statistically significant as demonstrated by the unpaired two tailed Student's *t-*test (Figure 6E). Thus, ANK most likely potentiates MP movement through PD by reducing PD callose deposits, and this down-regulation of the callose content requires the presence of both ANK and MP.

## Discussion

The molecular mechanism by which MP gates PD for viral trafficking remains obscure even two decades after the discovery of this activity of MP [15]. This is mainly due to the lack of knowledge about the host factor(s) with which MP interacts during the PD gating process. Once such factor, actin, is suggested by a recent study showing that several viral MPs may sever actin filaments to relax PD during their cell-to-cell movement [25]. Here, we characterized another cellular factor, a tobacco cytoplasmic protein ANK, which likely facilitates MP transport through PD. Our data demonstrate direct interaction between ANK and MP *in vivo* and *in vitro* and indicate that this interaction results in reduction of callose deposits at PD and enhanced cell-to-cell movement of MP. Conversely, ANK deficit results in reduced movement of MP and slower spread of viral infection. Mechanistically, ANK promotes increase in PD transport by reducing callose deposits at PD and, by implication, relaxing the callose sphincter around the PD channel.

There are two interesting aspects to this activity of ANK. First, it binds and affects PD transport of MP, which is known to target specifically to PD and gate and traverse them [13], [15], [16]. On the other hand, it does not bind to or affect PD transport of proteins, such as CNX, ACA2 and free YFP, that do not specifically localize to PD and move through them presumably by simple diffusion which does not involve PD gating [53]. Thus, ANK most likely is involved in active PD gating, but not in mere endomembrane-mediated diffusion through PD. Second, ANK cannot elicit its effects on PD transport alone, but does so in concert with MP. Thus, MP does not simply use the existing PD transport pathway, but actively interacts with the host components and participates in the gating activity. MP, thought to associate with endomembranes [54], [55], [57], [70], interacts with ANK most likely through its cytoplasmic domains [56], [58]. Because MP specifically targets to PD [13], it may help direct ANK, a predominantly cytoplasmic protein, to PD.

ANK is a multifunctional protein, the roles of which in different plant species are just beginning to emerge. In Arabidopsis, there are two highly conserved ANK proteins, both of which are involved in chloroplast biogenesis by binding to its outer envelope membrane proteins and delivering them to their destination [45]. The Arabidopsis AKR2 also participates in ROS scavenging via interaction with ascorbate peroxidase [40]. Also, both Arabidopsis and tobacco ANKs are involved in disease resistance against *Pseudomonas syringe* [40], [41], and ANK/HBP1/TIP1 from the *N*-gene containing *N. tabacum* cv. Xanthi in hypersensitive cell death response to TMV [41]. ANK/HBP1/TIP1 and two ANKs from *N. tabacum* cv. Samsun, i.e., TIP2 and TIP3, may bind MP of PVX; these interactions, however, were shown only in the yeast two-hybrid system, and potential involvement of these ANKs in potexviral movement has not been explored [35]. Finally, also in the yeast two-hybrid system, ANK/HBP1/TIP1, TIP2 and TIP3 interact with vacuolar β-1,3-glucanases [35], [71]; because these ANKs and β-1,3-glucanases reside in different subcellular compartments, this interaction, presumably, is not biological [72]. Furthermore, all known β-1,3-glucanases that might degrade callose are predicted to be either vacuolar or secreted [28], [73], [74], [75], [76], [77], [78], [79], [80] and are not known to possess cytoplasmic domains. The predicted subcellular localization suggests that it is physically impossible for these enzymes to interact with cytoplasmic ANK. One possibility is that ANK may be crucially involved during the targeting of β-1,3-glucanases containing ER-derived vesicles by MP to PD, as proposed recently [3], [53], by directly binding to MP. Another possible target for the ANK activity is cellular callose synthases. Callose is presumed to have a rapid turnover [81], and active callose synthases are likely critical to maintain the PD callose deposits. These enzymes usually contain several subunits, many of which have not been sufficiently characterized; however, at least some of them have been predicted to represent transmembrane proteins with cytoplasmic domains [82], [83], [84]. This topology of callose synthases would allow interactions with ANK and/or ANK- MP complexes. Regardless of its potential downstream targets in the molecular pathway of PD gating, ANK most likely represents a cellular factor that recognizes MP and acts synergistically with it to gate PD and mediate MP transport through these channels.

## Methods

### *ANK* cDNA

Total RNA was extracted from the *N. tabacum* cv. Turk plants by Tri-Reagent (Ambion), and its cDNA was synthesized using the RevertAid First Strand cDNA Synthesis Kit (Fermentas) with the oligo-dT primer. The full-length *ANK* cDNA was obtained by PCR using the primer pair 5′GCGAATTCTATGTCTGAGGGAGAGAAAGTTTTGCC3′/5′TCCCCCGGGTCACAGAAACACATCTTTCTCGAGCAG3′ and the total cDNA as template.

### Vectors for generation of transgenic plants and expression of untagged ANK and MP

For pANK, the PCR-amplified *ANK* cDNA was inserted into the EcoRI-SmaI sites of pSAT4A-35SP-MCS-35ST [85]. For pRNAi-ANK, the *ACTIN* intron 11 sequence from pSK-int [86] was first inserted into the HindIII-EcoRI sites of pSAT4A-35SP-MCS-35ST, resulting in pRNAi. To suppress the *ANK* gene specifically, we designed RNAi constructs that target the unique *ANK* cDNA sequences located between positions 1 and 406. These sequences of *ANK* were obtained from pANK by digesting it with BamHI-HindIII and BamHI-EcoRI and cloned into the BglII-HindIII and EcoRI-BamHI sites, respectively, of pRNAi. The resulting pRNAi-ANK construct carried the inverted repeat of the first 406 base pairs of the *ANK* cDNA, flanked by the *ACTIN* intron 11 sequences.

The entire expression cassettes from pRNAi-ANK and pANK were then cloned as I-SceI fragments into the binary vector pRCS2-nptII, which contains a kanamycin resistance selection marker [87], resulting in pRCS-RNAi-ANK and pRCS-ANK, respectively.

For pTMV MP, the MP coding sequence was PCR-amplified using the primer pair 5′AAGAATTCATGGCTCTAGTTGTTAAAGGAAAAGTG3′/5′TGCATCCCGGGTTAAAACGAATCCGATTCGGCGACAGT3′ and pTMV004 [88] as template. The amplified product was digested with the EcoRI and SmaI and inserted into the same sites of pSAT4A-nosP-MCS-nosT, which was constructed by transferring the nosP-MCS-nosT expression cassette as an AgeI-NotI fragment of pSAT2A-nosP-MCS-nosT [85] into the same sites of pSAT4-EGFP-C1 [87], replacing the EGFP-C1 expression cassette. Then, the expression cassette from pTMV MP was cloned as an I-SceI fragment into pRCS2-nptII, resulting in pRCS-TMV MP.

### Vectors for movement assay, subcellular localization, and BiFC

To construct pCFP-ANK and pnYFP-ANK, the PCR-amplified *ANK* cDNA was digested with EcoRI and SmaI, and the resulting fragment cloned into the same sites of pSAT4-ECFP-C1 and pSAT4-nEYFP-C1 [61], respectively. pSAT4-ECFP-C1 was made by transferring the ECFP-C1 expression cassette as an AgeI-NotI fragment of pSAT6-ECFP-C1 [87] into the same sites of pSAT4-EGFP-C1 [87], replacing the EGFP-C1 expression cassette. Then, the expression cassettes from pCFP-ANK and pnYFP-ANK were cloned as I-SceI fragments into pRCS2-nptII, resulting in pRCS-CFP-ANK and pRCS-nYFP-ANK.

For pTMV MP-YFP and pTMV MP-cYFP, the TMV MP-encoding sequence was first PCR-amplified using the primer pair 5′AAGAATTCATGGCTCTAGTTGTTAAAGGAAAAGTG3′/5′GGCCGGTACCGAAACGAATCCGATTCGGCGACAGT3′ and pTMV004 as template. The amplified fragment was digested with EcoRI and KpnI, inserted into the EcoRI-XbaI sites of pSAT4A-nosP-MCS-nosT by double-ligation with the YFP and cYFP coding sequences excised with KpnI-XbaI from pSAT4-EYFP-N1 and pSAT4-cEYFP-N1 [61], respectively. pSAT4-EYFP-N1 was made by transferring the EYFP-N1 expression cassette as an AgeI-NotI fragment of pSAT6-EYFP-N1 [87] into the same sites of pSAT4-EGFP-C1 [87], replacing the EGFP-C1 expression cassette. Then, the expression cassettes from pTMV MP-YFP and pTMV MP-cYFP were cloned as I-SceI fragments into pRCS2-nptII, resulting in pRCS-TMV MP-YFP and pRCS-TMV MP-cYFP.

For pnYFP-NADK3, *NADK3* cDNA sequence was obtained by PCR using primers 5′GCGAATTCATGGCGATTAGGAAGCTTTTGCTTCTTTTG3′/5′CATCCCGGGCTAGTACCTTGATCTGATCTGAG3′ and Arabidopsis cDNA library [89] as a template. The PCR-amplified *NADK3* cDNA was digested with EcoRI and SmaI, and the resulting fragment was cloned into the same sites of pSAT4-nEYFP-C1 [61].

Then, the expression cassette from pnYFP-NADK3 was cloned as an I-SceI fragment into pRCS2-nptII, resulting in pRCS-nYFP-NADK3.

For pCNX-YFP and pACA2-YFP, the *CNX* and *ACA2* cDNAs were amplified from the Arabidopsis cDNA library [89] using the respective sets of primers, 5′GCGAATTCTATGAGACAACGGCAACTATTTTCCG3′/5′GGCCGGTACCATTATCACGTCTCGGTTGCCTTTTGC3′, and 5′GCGAATTCTATGGAGAGTTACCTAAACGAGAAT3′/5′GGCCGGTACCAACGGGAATCGTCTTCAGTCCAGCG3′. The amplified products were digested with EcoRI and KpnI, and cloned into the same sites of pSAT4A-YFP-N1.

The PDCB-mCherry expression construct was kindly provided by Dr. Maule [52].

### Vectors for renatured gel blot overlay assay

The StrepII coding sequence [59] was included in the reverse primer of the primer pair 5′ GCGAATTCTATGTCTGAGGGAGAGAAAGTTTTGCC 3′/5′GCGGCCGCTTATTTTTCAAACTGCGGATGGCTCCAGGTACCCAGAAACACATCTTTCTCGAGCAG3′ and pANK as template. The StrepII coding sequence was also included in the reverse primer of the primer pair 5′GCGAATTCATGGCGATTAGGAAGCTTTTGCTTCTTTTG3′/5′GCGGCCGCTTATTTTTCAAACTGCGGATGGCTCCAGTACCTTGATCTGATCTGAGA3′ and used to amplify the *NADK3* cDNA from the pnYFP-NADK3. The amplified products were digested with EcoRI and EagI and inserted into the same sites of pET28c(+) (Novagen), producing pET28-ANK-StrepII and pET28-NADK3-StrepII, respectively.

To produce GST-MP, the MP coding sequence was excised from pTMV MP by digesting it with EcoRI and SmaI inserted into the same sites of pGEX-5X-1 (GE Healthcare Life Sciences), producing pGEX-TMV MP.

All PCR reactions were performed using a high-fidelity proofreading *Pfu* Turbo DNA polymerase (Stratagene), and their products were verified by DNA sequencing using an Applied Biosystems 3730 Genetic Analyzer.

### Generation of transgenic plants with suppressed or enhanced ANK expression

pRCS-RNAi-ANK and pRCS-ANK were introduced into the disarmed *Agrobacterium* strain EHA105, which was then used to transform *N. tabacum* cv. Turk as described [90]. The resulting transgenic plants were selected on kanamycin-containing media, and maintained according to our standard protocol [22]. The transgenic plants were vegetatively propagated, and the suppression or overexpression of *ANK* in the ANK and RNAi ANK lines, respectively, was monitored by reverse transcription (RT) followed by quantitative (q) PCR. Control, wild-type tobacco plants were grown as described [22]. All plants were transferred to soil and grown in an environment-controlled chamber at 22–24°C under long day conditions of 16 h white light (70–80 µmol photons m-2 s-1) and 8 h dark. All experiments utilized 5–6-week-old plants with 6–8 leaves.

### RT-PCR and qPCR

Total RNA was extracted from tissue samples using Tri-Reagent (Ambion), treated with RNase-free DNase I (Fermentas), and 0.5-µg samples of the resulting preparations were reverse-transcribed using the RevertAid First Strand cDNA Synthesis Kit (Fermentas), according to the manufacturer's instructions. RT reaction products were amplified by qPCR in a 7300 Real-Time PCR System (Applied Biosystems) with Maxima™ SYBR Green qPCR Master Mix (Fermentas) using primer pairs specific for *ANK* (5′AGGCTGCACTAACTGCTGGT3′/5′TTACAGCGGCTCCATTCTCT3′), *MP* (5′AAAGATTTCAGTTCAAGGTCGTTCC 3′/5′TCCGTCTCTCACGTTTGTAATCTTC3′), *ACTIN* (5′TCACTGAAGCACCTCTTAACC3′/5′CAGCTTCCATTCCAATCATTG3′), and *TUBLIN*(5′TACACAGGGGAAGGAATGG/CTCGAAACCAACGCTTATC3′).

Relative abundance of the *ANK* or *MP* mRNA-specific products was normalized to the amount of the product specific for *ACTIN* and *TUBLIN*, respectively, which represented an internal control of a constitutively expressed gene. The absence of potential residual genomic DNA contamination was confirmed by control qPCR reactions performed without RT.

### Agroinfiltration and microbombardment

For agroinfiltration, binary vectors were introduced into the *Agrobacterium* EHA105 strain as described [91], grown overnight at 28°C, and infiltrated into intact *N. tabacum* leaves as described [92], [93]. Microbombardment experiments were performed as described [46]. Briefly, 100 µg of DNA preparations was adsorbed onto 10 mg of 1-µm gold particles, which were then microbombarded into the lower leaf epidermis at a pressure of 80–110 psi, using a portable Helios gene gun system (Model PDS-1000/He, Bio-Rad). All experiments were repeated at least three times.

### Cell-to-cell movement assay

Leaves with the length of 18 cm, excluding petiole, from 4–5 week-old plants were selected for all experiments. Constructs expressing YFP-tagged tested proteins, i.e., pTMV MP-YFP, pCNX-YFP and pACA2-YFP, were microbombarded into the lower epidermis at the equivalent locations on each leaf. When movement of two proteins was compared, their expression constructs were introduced at the symmetrical locations relatively to the mid-rib of the leaf. At least 120 YFP-expressing clusters in each microbombarded tissue were observed under a Zeiss LSM 5 Pascal confocal microscope. After 24 h, all expression clusters were represented by single cells and considered to indicate the absence of movement. After 48 h, the number of fluorescent cells in each expression cluster varied due to cell-to-cell movement. At this time, the number of cells in each expression cluster was recorded, followed by statistical evaluation of resulting data by the unpaired two-tailed Student's *t-*test. Differences between sets of measurements with p-values less than 0.001, corresponding to the statistical probability of greater than 99.9%, were considered statistically significant.

### TMV infection assay

pTRBO-DsRed (kindly provided by Dr. Jens Tilsner, University of Edinburgh) was agroinfiltrated into 18-cm, excluding petiole, leaves from 4–5 week-old plants. Four to 14 days after inoculation, at least 40 DsRed-expressing clusters in each infiltrated tissue were analyzed by confocal microscopy, followed by statistical evaluation of the DsRed-expressing surface area measurements by the unpaired two-tailed Student's *t-*test. Differences between sets of measurements with *P*-values <0.001, corresponding to the statistical probability of greater than 99.9%, were considered statistically significant.

### Protoplast preparation and TMV RNA replication assay

Protoplasts were prepared from the 3–8-cm, excluding petiole, leaves from 4–5 week old plants. The leaves were sterilized in 50% bleach with 0.1% SDS for 5 min, followed by 5 rinses in distilled water and overnight incubation at room temperature in the protoplasting enzyme mixture (40 mg/ml cellulase Onozuka R-10 (Phytotechnology Laboratories) and 1.5 mg/ml macerozyme (MP Biomedicals) in 500 mM D-sorbitol, 1 mM CaCl_2_, 5 mM MES, pH 5.5). The protoplasts were collected by centrifugation at 600 x g for 5 min, resuspended in the MMC buffer (0.7 M mannitol, 10 mM CaCl_2_, 5 mM MES, pH 5.8) at 2×10^7^ cells/ml. For transformation with the viral infectious clone, 5 ml of 30% PEG 8000 in the MMC buffer and 4 µg of the pTRBO-DsRed DNA were added to 2 ml of protoplast suspension and mixed gently. After 15-min incubation at room temperature in the dark, 40 ml of the culture medium (3% sucrose, 500 mM D-mannitol, MS salts, 5 mM MES, pH 5.7) was added to the protoplast suspension. The incubation was continued for an additional 45 min, after which the protoplasts were collected by centrifugation, resuspended in 35 ml of the culture medium, and incubated further to allow replication of the virus. Protoplast samples (5 ml) were harvested 16, 28, 40, 52, and 64 h after transformation, and their total RNA was extracted by the TRIzol (Invitrogen) method according to the manufacturer's instructions.

### Subcellular localization and BiFC

For subcellular localization, pTMV MP-YFP and pCFP-ANK were expressed in tobacco leaves following microbombardment, and the subcellular fluorescence pattern was analyzed by confocal microscopy. For BiFC, pRCS-nYFP-ANK and pRCS-TMV MP-cYFP, or pRCS-nYFP-ANK and pRCS-nYFP-NADK3 binary constructs were expressed in tobacco leaves following agroinfiltration, and BiFC was detected by confocal microscopy as described [61]. For the combination of pRCS-nYFP-ANK and pRCS-TMV MP-cYFP, we used Agrobacterium cells at OD_600_ = 0.0015. For negative controls, bacterial cultures at OD_600_ of up to 0.6 were used to confirm the absence of reconstructed YFP signal even at very high inocula.

### Renatured gel blot overlay assay

Recombinant GST-MP, unfused GST, ANK-StrepII, and NADK3-StrepII were produced as described [94] in BL21(DE3) *Escherichia coli* strain (Novagen) from the pGEX-TMV MP, pGEX-5X-1, pET28-ANK-StrepII and pET28-NADK3-StrepII vectors. The identity of these proteins was confirmed by western blot analysis using anti-GST (Santa Cruz) and anti-StrepII (Genscript) polyclonal antibodies, and the amounts of these proteins in the total bacterial extracts were estimated by scanning densitometry of the corresponding bands on SDS-polyacrylamide gels stained with Coomassie Brilliant Blue R-250, using the known amounts of BSA as standard. Protein extracts containing 1 µg of GST-MP or unfused GST were resolved on 15% SDS-polyacrylamide gels, followed by eletrotransfer to a nitrocellulose membrane. The membrane-immobilized proteins were incubated with 0.5 µg/ml of ANK-StrepII or NADK3-StrepII and processed as described [32]. Binding of the tested proteins to the immobilized proteins was detected by probing the membranes with anti-StrepII rabbit polyclonal antibody (Genscript), followed by anti-rabbit IgG+M secondary antibody conjugated to HRP.

### Detection of ROS

Three days after agroinfiltration with binary constructs expressing the tested proteins, i.e., pRCS-ANK and/or pRCS-TMV MP, leaf disks from the agroinfiltrated areas were excised and transferred to the 10 mM MES (pH 5.6) containing SIGMA*FAST* 3,3′-diaminobenzidine (DAB) (Sigma-Aldrich). DAB was vacuum-infiltrated into the tissues for 20 min, followed by incubation of the leaf disks in the DAB staining solution for 4 h in the light and 16 h in the dark. After four brief washes in double-distilled water, the stained leaf disks were transferred to 95% ethanol and heated at 55°C to remove chlorophyll. The tissue was rehydrated, and imaged using an EPSON Perfection 4490 photo scanner.

### Whole-mount callose staining

Three days after agroinfiltration with pRCS-ANK and/or pRCS-TMV MP, tissue samples (<2×5 mm) from the agroinfiltrated areas were fixed by vacuum infiltration with 4% paraformaldehyde in the PIPES buffer (100 mM PIPES, 5 mM EGTA, 2 mM MgCl_2_, pH 6.9) followed by overnight incubation at 25°C with gentle agitation. The samples were then washed twice in double-distilled water and depleted of chlorophyll by incubation for 2 h at 25°C with gentle agitation in a graded series of ethanol (25, 50, 75, and 95%). For permeabilization, the dehydrated samples were air-dried, transferred to the PIPES buffer, and freeze-shattered as described [95]. Finally, the samples were transferred to a microscope slide, blocked in 1% BSA in PBS, and reacted with 1/200 dilution of anti-callose mouse monoclonal antibody (Biosupplies, Parkville, Australia) in 1% BSA in PBS, followed by the Alexa-488-conjugated anti-mouse IgG+M secondary antibody (Jackson ImmunoResearch, 1/200 dilution in 1% BSA in PBS). After three 10-min washes in PBS, the immunostained samples were mounted on microscope slide, using BioMount (Electron Microscopy Sciences), with the abaxial side of the leaf facing up and observed under a confocal microscope. To avoid measuring callose induced by the wounding made by the initial step of the sample preparation, the section in the middle of the tissue piece was selected for the observation. For each experiment, the confocal microscopy analysis included five samples immunostained independently. The total signal intensity in a 318×318 µm-area was measured using the fluorescence intensity quantification function of the LSM 5 Pascal software for least five fields per sample or a total 25 fields per experiment. To measure background signal intensities, mock-transformed samples, i.e., tissues infiltrated with Agrobacterium carrying empty expression vector, were immunostained in the absence of the primary antibody. These background signal values were subtracted from the signal values obtained from the samples, the average signal intensities with standard error for the each experiment were calculated and evaluated statistically by the unpaired two-tailed Student's *t-*test as described for analyses of the cell-to-cell movement data.

### Experimental conditions for the supplemental figures

All the procedures for the experiments documented in Figures S1, S2, S3, S4 and S5 and Table S1 are presented in File S1(Supplemental Methods).

### Accession numbers

The GenBank accession number for the *ANK* sequence reported in this paper is GU320195. The accession numbers for the ANK homologs are: *Nt*ANK1/HBP1 from *N. tabacum* cv. Xanthi; AAK18619/AAN63819, *Nt*TIP2 and *Nt*TIP3 from *N. tabacum* cv. Samsun; AAO91861.1 and AAO91862.1, *At*AKR2B and *At*AKR2 from Arabidopsis thaliana; NP_179331 and NP_849498. The accession numbers for the two genes that contains 17-bp identical to the sequence found in *ANK* are: *N. tabacum AATF*; AB126259.1, and *MgPP*; AF014052.1.

## Supporting Information

Table S1 Relative expression levels of *N. tabacum ASPARTATE AMINOTRANSFERASE* (*AATF*) and *MAGNESIUM PROTOPORPHYRIN IX* (*MgPP*) genes in wild-type, RNAi ANK1 and RNAi ANK2 plants. ^A^The shown values were normalized to the amounts of *ACTIN* transcript in the same samples. ^B^WT, wild-type. ^C^Standard deviations are indicated. ^D^P-values for the sets of data obtained from the WT and RNAi ANK1 or RNAi ANK2 plants were calculated using the Student t-test. Note that they show no statistically significant differences between expression levels of the tested genes between the RNAi transgenic lines and the wild-type plants.
(0.03 MB DOC)

Figure S1 ANK cDNA sequence. The nucleotide sequence encoding the ankyrin repeat-containing domain (positions 670–1032) is shadowed, and the sequence used for the pRNAi-ANK construct is underlined. The segment used for pRNAi-ANK is unique to ANK, except for the 17-bp region highlighted in black (positions 19–45), which is found in two *N. tabacum* genes, aspartate aminotransferase (AB126259.1) and magnesium protoporphyrin IX (AF014052.1). The sequences for primers used for qRT-PCR are also indicated. As these primers are designed to detect the sequence specifically conserved in the ANK family, but not found in other ankyrin repeat-containing proteins, qRT-PCR using this primer set allows specific quantification of all close homologs of ANK.
(1.10 MB TIF)

Figure S2 RNAi transgenic plants with severe and moderate suppression of *ANK* expression. (A) Quantification of the levels of *ANK* transcripts with indicated standard deviations. Plants with *ANK*. gene expression levels reduced to 5% and 15–40% of the wild-type expression level were designated severe and moderate suppressors, respectively. Plants were analyzed four weeks after their transfer to growth chamber from tissue culture. (B) Chlorotic leaf phenotypes in RNAi ANK3, but not in RNAi ANK1 or wild-type (WT) plants.
(1.21 MB TIF)

Figure S3 Altered *ANK* expression levels do not affect MP-YFP targeting to PD or protein expression. (A–F) PD localization of MP-YFP at 16 h (A–C) and 24 h (D–F) after bombardment in wild type plants (A, D), RNAi ANK2 (B, E), and ANK1 (C, F). Bars  = 20 µm. Plastid autofluorescence is in white. All images are single confocal sections. (G) MP-YFP expression levels in different plant lines. Lane1, wild type; lane 2, ANK1; lane 3, ANK2; lane 4, RNAi ANK1; lane 5, RNAi ANK2. Arrow indicates the position of MP-YFP.
(4.60 MB TIF)

Figure S4 Positive correlation between ANK-StrepII transcripts and protein levels. (A) Western blot analysis of *ANK-StrepII* in extracts prepared from the independent transgenic lines ANK-StrepII 1 to 6. (B) qRT-PCR analysis of the ANK-StrepII mRNA levels in the same transgenic lines. The shown values were normalized to the amounts of *ACTIN* transcript in the same samples.
(1.53 MB TIF)

Figure S5 Histochemical detection of ROS. The leaf discs were excised from the indicated plant lines and treated with DAB. No significant difference in ROS levels were observed between plant lines with different expression levels of ANK. WT; wild type. RNAi1 and 2; RNAi ANK1 and RNAi ANK2, respectively.
(2.62 MB TIF)

File S1 Supplemental Methods
(0.02 MB DOC)
