# Supplementary material for: ANK, a Host Cytoplasmic Receptor for the Tobacco mosaic virus Cell-to-Cell Movement Protein, Facilitates Intercellular Transport through Plasmodesmata
Source: PLoS Pathog. 2010 Nov 18;6(11):e1001201. doi: 10.1371/journal.ppat.1001201 (PMC2987828; doi:10.1371/journal.ppat.1001201)
Supplement: Table S1 — Relative expression levels of N. tabacum ASPARTATE AMINOTRANSFERASE (AATF) and MAGNESIUM PROTOPORPHYRIN IX (MgPP) genes in wild-type, RNAi ANK1 and RNAi ANK2 plants. AThe shown values were normalized to the amounts of ACTIN transcript in the same samples. BWT, wild-type. CStandard deviations are indicated. DP-values for the sets of data obtained from the WT and RNAi ANK1 or RNAi ANK2 plants were calculated using the Student t-test. Note that they show no statistically significant differences between expression levels of the tested genes between the RNAi transgenic lines and the wild-type plants. (0.03 MB DOC) [file ppat.1001201.s001.doc]

**Supplemental Table 1**

Relative expression levels of *N. tabacum* *aspartate aminotransferase* (*AATF*) and *magnesium protoporphyrin IX* (*MgPP*) genes in wild-type, RNAi ANK1 and RNAi ANK2 plants

|  | ^A^*AATP and MgPP* mRNA levels | | |
| --- | --- | --- | --- |
|  | ^B^WT | RNAi ANK1 | RNAi ANK2 |
| *AATF* | 5.68 ± ^C^0.22 | 6.24 ± 1.94  ^D^(*p*>0.1) | 5.62 ± 1.10  (*p*>0.1) |
| *MgPP* | 9.32 ± 4.4 | 11.7 ± 5.03  (*p*>0.1) | 7.49 ± 2.02  (*p*>0.1) |

^A^ The shown values were normalized to the amounts of *ACTIN* transcript in the same samples.

^B^ WT, wild-type.

^C^ Standard deviations are indicated

^D^ P-values for the sets of data obtained from the WT and RNAi ANK1 or RNAi ANK2 plants were calculated using the Student *t-*test. Note that they show no statistically significant differences between expression levels of the tested genes between the RNAi transgenic lines and the wild-type plants.
