# Supplementary figures and images for: ANK, a Host Cytoplasmic Receptor for the Tobacco mosaic virus Cell-to-Cell Movement Protein, Facilitates Intercellular Transport through Plasmodesmata
Source: PLoS Pathog. 2010 Nov 18;6(11):e1001201. doi: 10.1371/journal.ppat.1001201 (PMC2987828; doi:10.1371/journal.ppat.1001201)

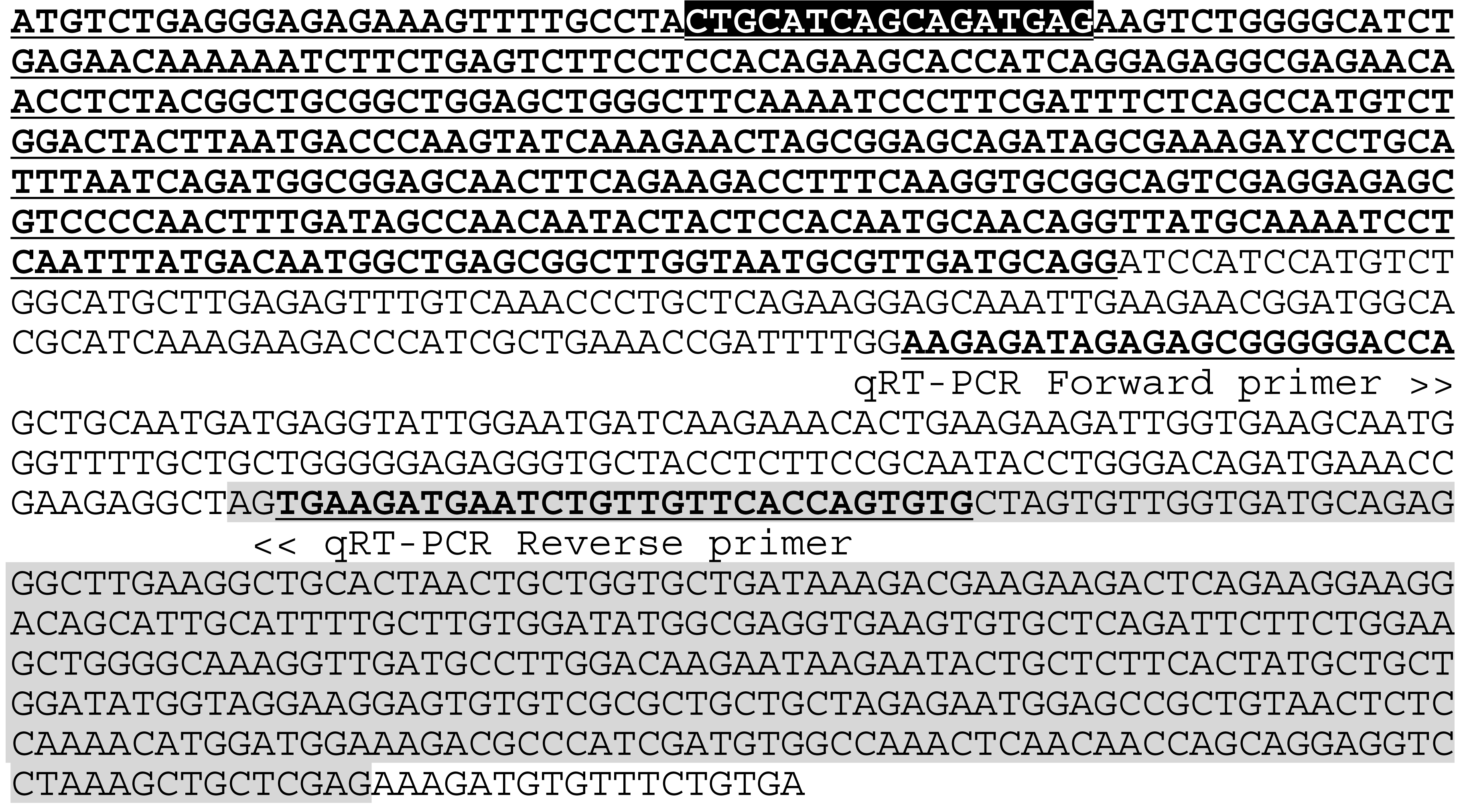

Supplement: Figure S1 — ANK cDNA sequence. The nucleotide sequence encoding the ankyrin repeat-containing domain (positions 670–1032) is shadowed, and the sequence used for the pRNAi-ANK construct is underlined. The segment used for pRNAi-ANK is unique to ANK, except for the 17-bp region highlighted in black (positions 19–45), which is found in two N. tabacum genes, aspartate aminotransferase (AB126259.1) and magnesium protoporphyrin IX (AF014052.1). The sequences for primers used for qRT-PCR are also indicated. As these primers are designed to detect the sequence specifically conserved in the ANK family, but not found in other ankyrin repeat-containing proteins, qRT-PCR using this primer set allows specific quantification of all close homologs of ANK. (1.10 MB TIF) [file ppat.1001201.s002.tif]

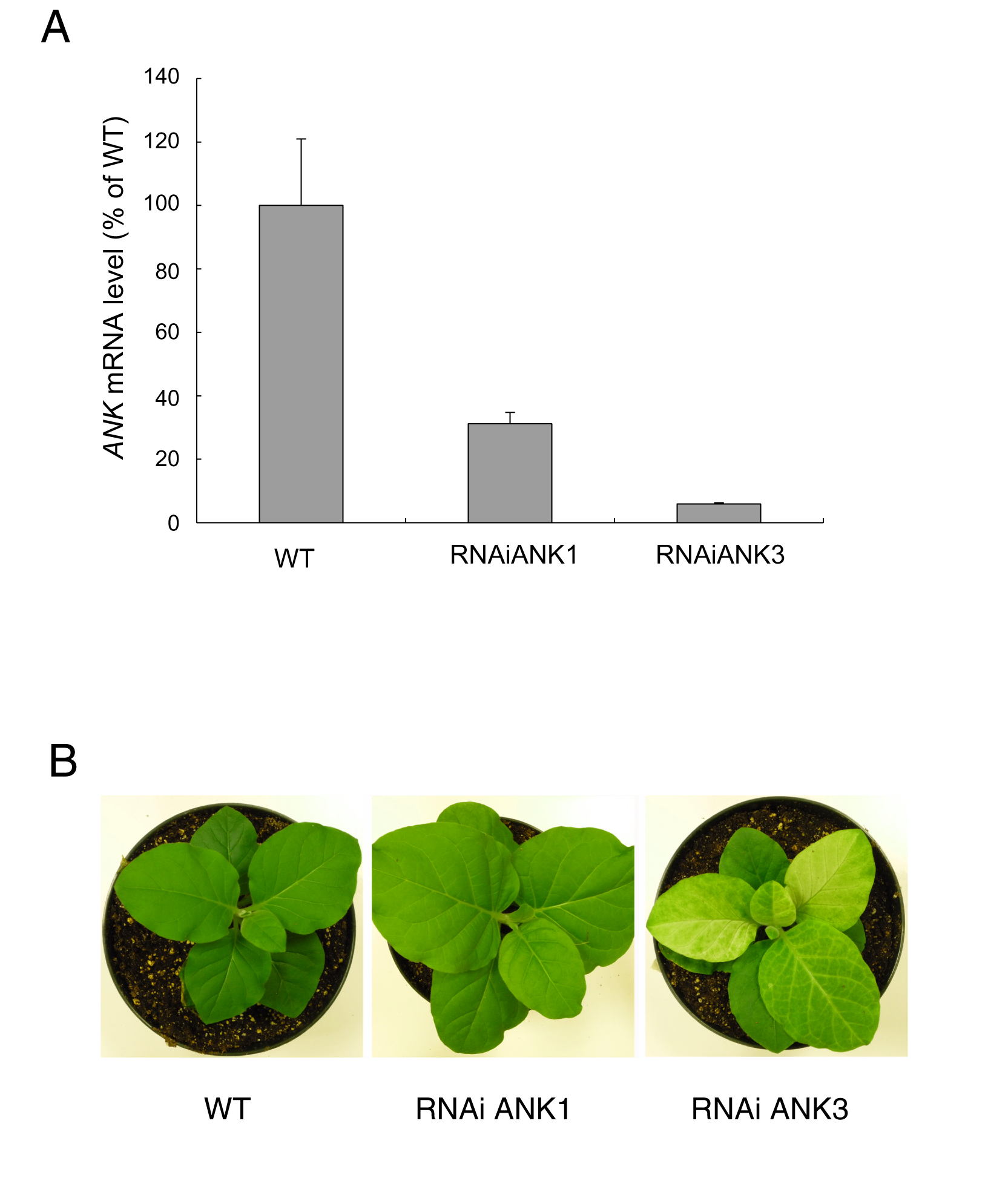

Supplement: Figure S2 — RNAi transgenic plants with severe and moderate suppression of ANK expression. (A) Quantification of the levels of ANK transcripts with indicated standard deviations. Plants with ANK. gene expression levels reduced to 5% and 15–40% of the wild-type expression level were designated severe and moderate suppressors, respectively. Plants were analyzed four weeks after their transfer to growth chamber from tissue culture. (B) Chlorotic leaf phenotypes in RNAi ANK3, but not in RNAi ANK1 or wild-type (WT) plants. (1.21 MB TIF) [file ppat.1001201.s003.tif]

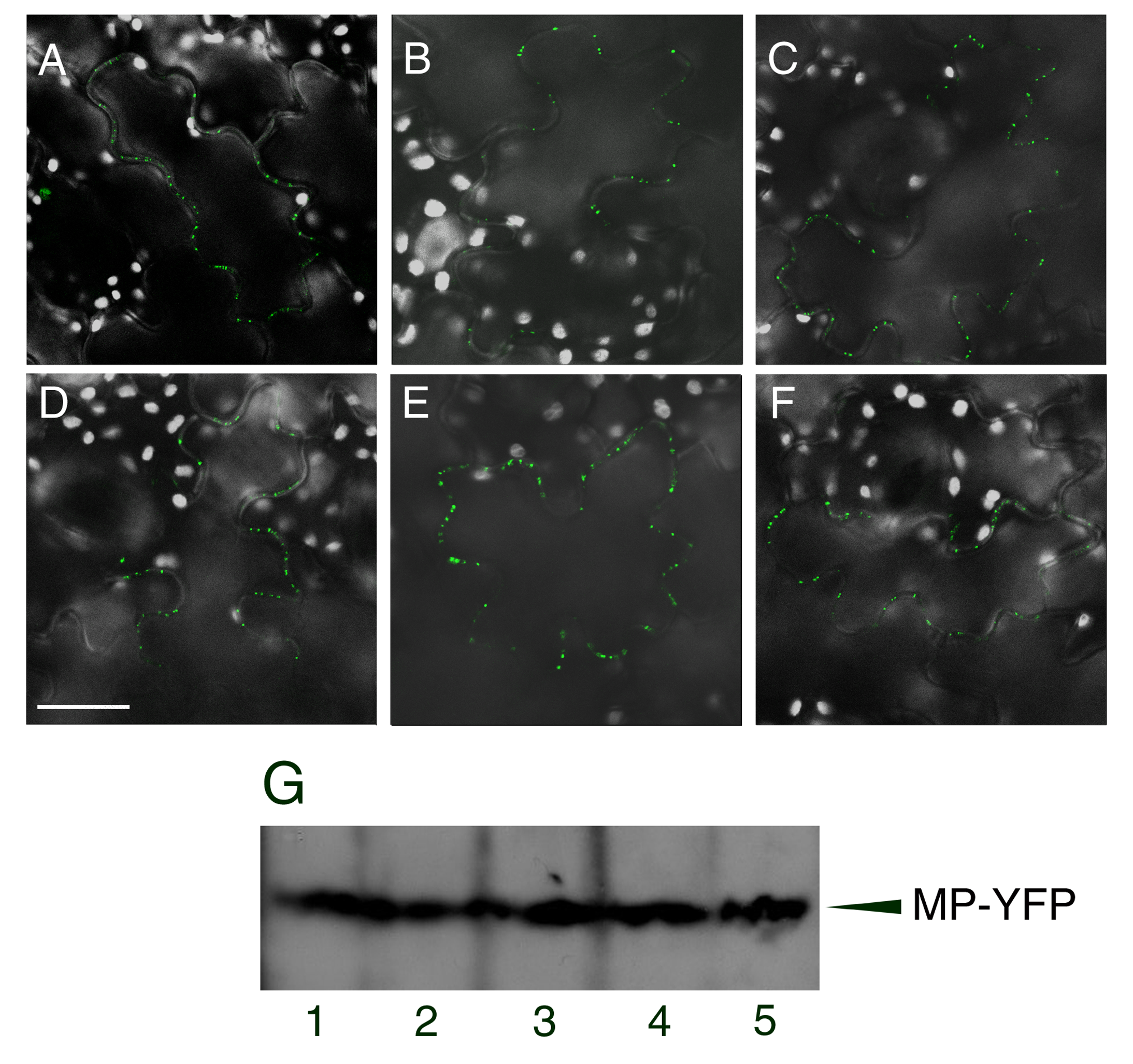

Supplement: Figure S3 — Altered ANK expression levels do not affect MP-YFP targeting to PD or protein expression. (A–F) PD localization of MP-YFP at 16 h (A–C) and 24 h (D–F) after bombardment in wild type plants (A, D), RNAi ANK2 (B, E), and ANK1 (C, F). Bars = 20 µm. Plastid autofluorescence is in white. All images are single confocal sections. (G) MP-YFP expression levels in different plant lines. Lane1, wild type; lane 2, ANK1; lane 3, ANK2; lane 4, RNAi ANK1; lane 5, RNAi ANK2. Arrow indicates the position of MP-YFP. (4.60 MB TIF) [file ppat.1001201.s004.tif]

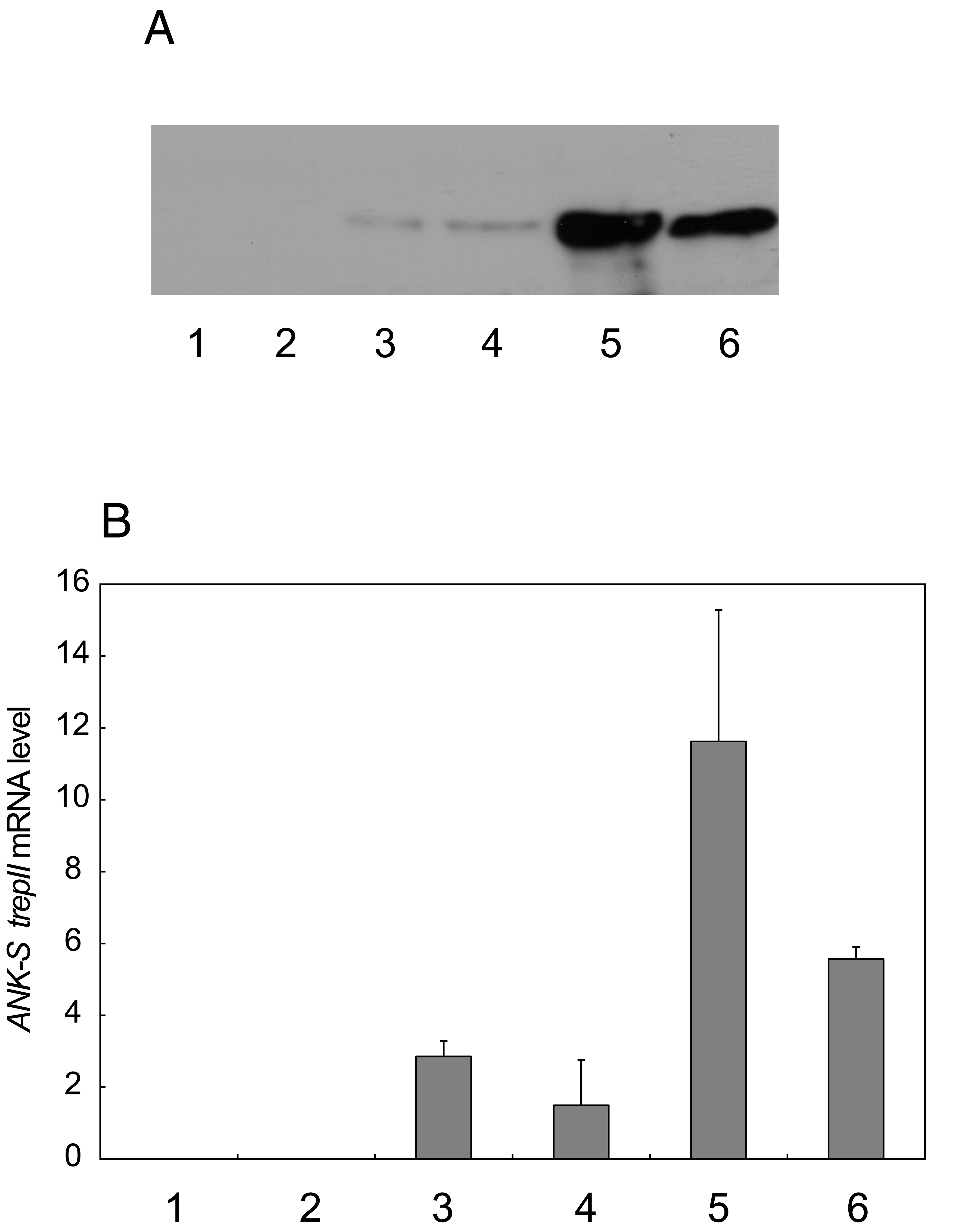

Supplement: Figure S4 — Positive correlation between ANK-StrepII transcripts and protein levels. (A) Western blot analysis of ANK-StrepII in extracts prepared from the independent transgenic lines ANK-StrepII 1 to 6. (B) qRT-PCR analysis of the ANK-StrepII mRNA levels in the same transgenic lines. The shown values were normalized to the amounts of ACTIN transcript in the same samples. (1.53 MB TIF) [file ppat.1001201.s005.tif]

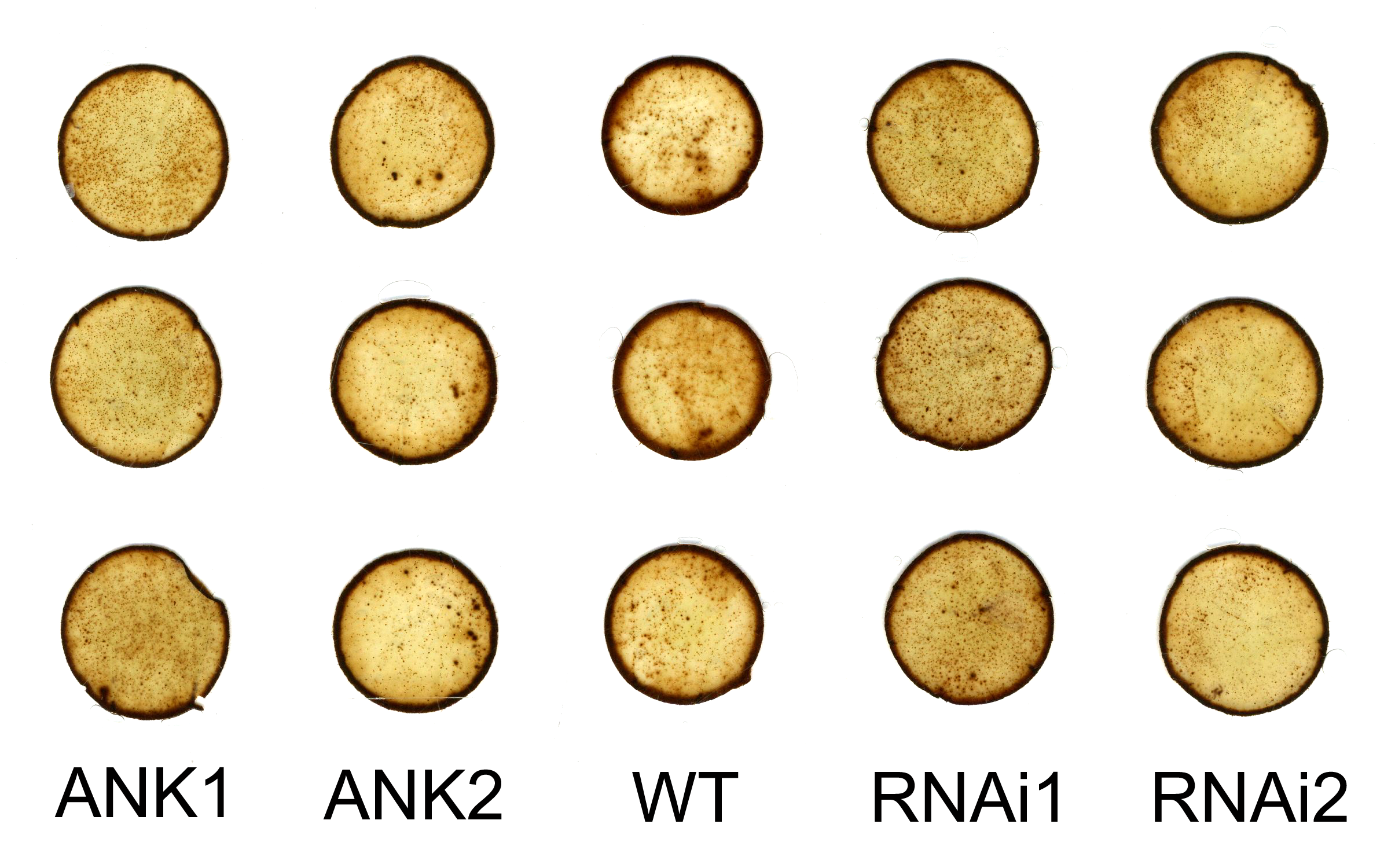

Supplement: Figure S5 — Histochemical detection of ROS. The leaf discs were excised from the indicated plant lines and treated with DAB. No significant difference in ROS levels were observed between plant lines with different expression levels of ANK. WT; wild type. RNAi1 and 2; RNAi ANK1 and RNAi ANK2, respectively. (2.62 MB TIF) [file ppat.1001201.s006.tif]
