# Supplementary material for: ANK, a Host Cytoplasmic Receptor for the Tobacco mosaic virus Cell-to-Cell Movement Protein, Facilitates Intercellular Transport through Plasmodesmata
Source: PLoS Pathog. 2010 Nov 18;6(11):e1001201. doi: 10.1371/journal.ppat.1001201 (PMC2987828; doi:10.1371/journal.ppat.1001201)
Supplement: File S1 — Supplemental Methods (0.02 MB DOC) [file ppat.1001201.s007.doc]

**Supplemental Methods**

**Detection of the MP-YFP protein in leaf tissues**. Leaf tissues transiently expressing MP-YFP following agroinfiltration were harvested, snap-frozen and ground to powder in liquid nitrogen. Then, 100-mg samples were incubated for 2 h at room temperature with gentle agitation in the 400 µl of protoplasting enzyme mixture (see Methods). The 100 µl of the SDS-PAGE loading buffer was added, and the mixture was boiled for 10 min to solubilize the protein, and 40-µl samples were analyzed by western blotting. MP-YFP was detected by probing the membranes with anti-GFP rabbit polyclonal antibody (Clontech), followed by anti-rabbit IgG+M secondary antibody conjugated to horseradish peroxidase (HRP).

**Detection of the ANK-strepII protien in leaf tissues.** Leaf tissues from the ANK-strepII transgenic lines were harvested, snap-frozen and ground to powder in liquid nitrogen. Then, 100-mg samples was resuspended in the 400 µl of 100 mM potassium phosphate buffer pH 5.7, combined with 100 µl of the SDS-PAGE loading buffer, and boiled for 10 min to solubilize the protein. 40-µl samples were analyzed by western blotting using anti-StrepII rabbit polyclonal antibody (Genscript), followed by anti-rabbit IgG+M secondary antibody conjugated to HRP
